# Paget's Disease of the Spine as a Catalyst for Osteosarcoma: A Narrative Review

**DOI:** 10.7759/cureus.109625

**Published:** 2026-05-25

**Authors:** Gonzalo F Del Rio Montesinos, Kevin Yoon, Amanuel Ayano, Linus Lee, Mark Ehioghae, Jonathan P Japa, Lancelot A Benn, Addisu Mesfin

**Affiliations:** 1 Orthopedics, Universidad Central del Caribe, Bayamon, USA; 2 Orthopedics, MedStar Hospital Research Institute, Washington D.C., USA; 3 Orthopedics, Edward Via College of Osteopathic Medicine, Blacksburg, USA; 4 Orthopedics, MedStar Washington Hospital Center, Washington D.C., USA; 5 Orthopedics, MedStar Orthopedic Institute, Washington D.C., USA; 6 Orthopedic Surgery, MedStar Washington Hospital Center, Washington D.C., USA; 7 Orthopedic Spine Surgery, MedStar Washington Hospital Center, Washington D.C., USA

**Keywords:** malignant transformation, osteosarcoma, paget’s disease of bone (pdb), primary spine tumor, spine

## Abstract

Paget's disease of bone (PDB) is one of the most common metabolic bone diseases worldwide. The spine is one of the most common locations for Paget's disease; however, data regarding the incidence, demographic patterns, clinical presentation, and management outcomes of malignant transformation in this region remain limited. We conducted a narrative review to synthesize the existing data and identify knowledge gaps. We found that osteosarcoma arising from PDB of the spine is extremely rare, but when transformation does occur, it most commonly involves the lumbar spine. Pagetic osteosarcoma typically presents in older men. The most prevalent symptom in these cases is back pain, but neurologic issues are also common and can range from radiculopathy to myelopathy and autonomic dysfunction. In cases of PDB in which malignant transformation is suspected, magnetic resonance imaging or computed tomography should be performed in addition to a bone biopsy. Management should be multidisciplinary and individualized, with systemic therapy and surgery considered according to disease extent, surgical feasibility, and patient factors. Outcomes for spinal osteosarcoma secondary to PDB are unfavorable, with past studies reporting a five-year survival rate as low as 10%. En bloc surgical resection may offer a greater chance of survival, but this is not always technically achievable given the surrounding anatomic constraints. Palliative care, physical medicine, and rehabilitation also play important roles in supporting patients through complex spine surgical treatment. Malignant transformation into osteosarcoma is a rare complication of PDB, of which spine surgeons should be aware. Like other forms of spinal osteosarcoma, this condition is challenging to treat and carries a poor prognosis. Patients should be referred to centers with multidisciplinary experience in the management of spinal sarcomas. Significant gaps remain in risk stratification, surveillance, and treatment algorithms for pagetic osteosarcoma of the spine, where data are limited. Standardized, multicenter studies are needed to improve prognostication and management.

## Introduction and background

Paget's disease of bone (PDB), also known as osteitis deformans, is among the most common metabolic bone disorders worldwide and is characterized by excessive osteoclastic bone resorption followed by compensatory, excessive, and disorganized osteoblastic bone formation [1]. Among individuals older than 75 years, the incidence of PDB has been reported to reach 5 per 10,000 person-years in men and 3 per 10,000 person-years in women [2,3]. PDB frequently affects the axial skeleton, with spinal involvement reported in up to 15.2% of cases [4,5]. When the spine is involved, progressive cortical thickening and trabecular disorganization may result in deformity, neurologic compromise, and mechanical instability.

One of the most serious recognized complications of PDB is malignant transformation, most commonly to osteosarcoma [6]. Huvos reported in 1983 that 27% of individuals older than 40 years with osteosarcoma had PDB as a predisposing condition [6]. Other studies have estimated malignant transformation rates ranging from 0.1% to 2% [7-10]. Although osteosarcoma is the predominant malignancy associated with PDB, other sarcoma subtypes have also been described, albeit less frequently [11,12]. Despite its rarity, malignant transformation in PDB represents a substantially increased risk compared with osteosarcoma in the general population, in whom it occurs in less than 0.1% of individuals [11].

Important gaps remain in understanding the mechanisms underlying pagetic osteosarcoma, particularly in the axial skeleton. Much of the existing literature consists of case reports and small retrospective series, limiting definitive conclusions regarding pathogenesis, risk stratification, and optimal management. Current evidence suggests that malignant transformation in PDB occurs in fewer than 1% of affected patients but still confers a markedly elevated risk of osteosarcoma relative to the general population [13-16]. Although PDB commonly involves the axial skeleton, malignant degeneration more often arises in the pelvis, femur, and humerus, with spinal transformation remaining comparatively uncommon [6,14,17-19].

Molecular mechanisms implicated in this process include SQSTM1 mutations with constitutive NF-κB activation, loss of heterozygosity on chromosome 18q, TP53 and RB1 pathway alterations, IL-6-mediated inflammatory signaling, and oncogenic mediators such as c-fos [13,20-38]. Clinically, pagetic osteosarcoma carries a poor prognosis, with survival outcomes substantially worse than those of sporadic osteosarcoma, particularly in older patients and those with axial disease [6,17,19,39,40].

Given the unique diagnostic and therapeutic challenges associated with spinal involvement, a focused synthesis of the available literature is warranted. This narrative review aims to summarize the epidemiology, molecular basis, clinical presentation, imaging characteristics, management strategies, and outcomes of malignant transformation of Paget's disease involving the spine, with particular emphasis on osteosarcoma.

## Review

Literature search

A comprehensive literature search was conducted to identify studies reporting malignant transformation of Paget's disease of bone (PDB) involving the spine. This manuscript was conducted as a narrative literature review. Electronic searches were performed in PubMed/MEDLINE and Embase from database inception through November 2025. The search strategy combined controlled vocabulary terms and free-text keywords related to Paget's disease of bone, malignant transformation, and spinal involvement. Search terms included combinations of "Paget's disease of bone" or "osteitis deformans", "osteosarcoma" or "sarcoma", and "spine", "vertebral", or "axial skeleton". Reference lists of included articles were manually reviewed to identify additional relevant studies not captured in the initial database search.

Study selection

The initial search yielded 565 records (PubMed n=475; Embase n=90). After removal of duplicates, 457 unique records remained. Titles and abstracts were screened independently by two reviewers in a two-stage selection process. After title and abstract screening, 179 articles were selected for full-text review.

Of these, 122 were excluded because they lacked primary patient data, did not involve spinal PDB, addressed non-malignant complications only, or did not provide sufficient clinical detail. Eleven articles required secondary review due to uncertainty regarding eligibility; these were resolved through consensus discussion between the reviewers. A final total of 57 studies met the inclusion criteria and were included in the qualitative synthesis.

Eligibility criteria

Studies were eligible for inclusion if they reported original patient data involving PDB affecting the spine and described malignant transformation, including osteosarcoma or other sarcomas. Only articles published in English were considered. Studies were excluded if they involved PDB without spinal involvement, addressed non-malignant complications exclusively, consisted of review articles without original patient data, were conference abstracts without accessible full text, or represented duplicate reporting of previously published cases.

Data extraction and synthesis

Data extracted from eligible studies included patient demographics, spinal level involved, histologic subtype of malignancy, presenting symptoms, imaging modalities used for diagnosis, treatment strategies, and reported outcomes. Given the rarity of malignant transformation in spinal PDB and the predominance of case reports and small case series, a qualitative synthesis was performed rather than a quantitative meta-analysis. Findings were summarized descriptively to identify recurring clinical patterns, diagnostic approaches, management strategies, and outcomes associated with spinal sarcomas arising in PDB.

Review of the literature

Given the narrative nature of this review, findings are presented thematically across key domains including epidemiology, risk factors, clinical presentation, imaging, and management.

Epidemiology

Osteosarcoma arising from PDB of the spine is extremely rare. For instance, when reviewing three large case series with a total of 227 patients with PDB, we found that only 12 cases of transformation within the spine were recorded [9,10,41]. Earlier reports from the mid-20th century series estimated rates of malignant transformation in Paget's disease to range between 1% and 2% [7-10]. More recent population-based studies have demonstrated a decline in transformation rates over the past several decades, with contemporary estimates as low as 0.2% [16,19,42]. When transformation does occur, it more frequently involves the lumbar spine than the thoracic or cervical spine [43-45].

**Figure 1:**
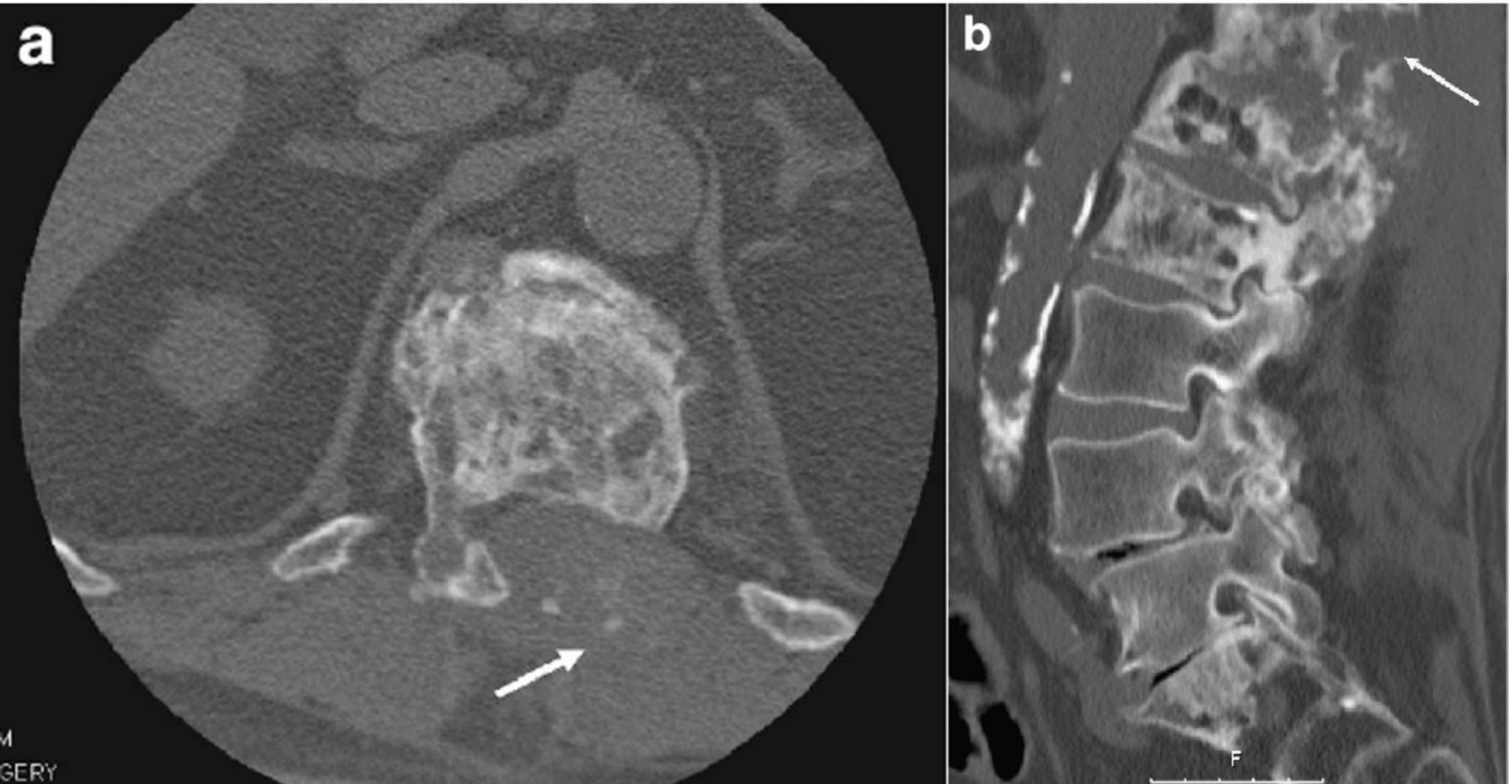
Computed tomography scan showing osteosarcoma in patient with history Paget's disease of bone (PDB) in the lumbar spine. Notice the extensive lytic pattern of destruction of the L1/2 vertebrae, which is pathologic for PDB. The arrows point toward an expanding mass that is the site of osteosarcoma secondary to PDB Reprinted from Sofka et al. [47], Copyright © 2006 by Sage Publications, with permission from Sage Publications.

Historically, degeneration into osteosarcoma was more common in patients with polyostotic forms of Paget's disease [3,10,12,46,47] (Figure 1). For instance, in a case series in which they reviewed radiographic findings of 22 patients with osteosarcoma secondary to PDB, Moore et al. found that 15 patients had a polyostotic form of Paget's disease; however, that series only included three cases of spinal PDB [48]. On the other hand, in a study of 37 cases of axial osteosarcoma, Deyrup et al. found no relationship between malignant transformation and polyostotic PDB, indicating that this association may not hold true for spinal PDB [40]. Interestingly, a non-malignant transformation into a giant cell tumor has been linked to a polyostotic, genetically linked form of PDB [49,50].

Typically, pagetic osteosarcoma presents in older men, with most patients first diagnosed after the age of 65 years; this is true for spinal osteosarcoma as well [7,9,14,40,48]. Interestingly, this presentation differs from global data on sporadic spinal osteosarcoma, which tends to occur more frequently in either adolescents or elderly patients, with an almost equivalent male-to-female ratio [8,51].

Outcomes for spinal osteosarcoma secondary to PDB are typically unfavorable, with past studies showing a five-year survival rate as low as 10% and a mean survival time ranging from 4.2 months to 27 months after diagnosis [7]. Comparatively, for patients older than 60 years with a diagnosis of osteosarcoma unrelated to PDB, the five-year survival rate has been found to be 24.2% without treatment [51]. Recent trials in which newer chemotherapy regimens were used have seen five-year survival rates jump as high as 60% for non-PDB-related osteosarcoma [52]. 

Fibrosarcoma, an abnormal proliferation of fibrous connective tissue, is also a malignancy secondarily associated with spinal PDB; however, these sarcomas account for less than 10% of those that arise in patients with PDB. These tumors typically present in a patient's seventh to eighth decade of life and have a slight male predominance (up to a 2:1 ratio) [7,18,40,53]. The outlook is similar to that of osteosarcoma, with studies showing five-year survival rates for fibrosarcoma secondary to PDB as low as 15% [7,53].

Risk stratification predictors of malignant transformation

Malignant transformation in Paget's disease of bone (PDB) remains rare, occurring in approximately 0.3-0.9% of affected individuals, yet it represents a clinically devastating complication [15,19,54,55]. Despite decades of recognition, reliable risk stratification models remain poorly defined. Current evidence suggests that malignant degeneration is influenced by disease burden, genetic susceptibility, and patient-specific clinical factors. 

Polyostotic disease and malignant risk

Historically, pagetic osteosarcoma was described as occurring predominantly in patients with long-standing, polyostotic disease. Early clinicopathologic analyses and clinical guidelines suggested that sarcomas "generally occur in patients with multiple bones involved" [14,18]. However, contemporary data challenge this assumption. A 2009 UK population-based study found that sarcoma occurred among polyostotic cases in the expected proportion, indicating that widespread skeletal involvement may not represent an independent risk factor for malignant transformation [19]. Notably, 46% of pagetic sarcoma cases in that series had monostotic disease [19]. Similarly, analysis from the Hamburg Bone Register reported malignant transformation in 0.8% of 754 PDB patients without demonstrating a clear association with disease extent [54]. These findings suggest that polyostotic involvement may reflect longer disease duration or ascertainment bias rather than a direct oncogenic driver.

Age and sex distribution

Advanced age remains the most consistent demographic risk factor. National Cancer Database data demonstrate a mean age at diagnosis of 68.9 years for pagetic osteosarcoma, with other series reporting median ages in the seventh to eighth decades[6,14,17,19]. This contrasts sharply with sporadic osteosarcoma, which predominantly affects adolescents and young adults. The 2009 UK series reported a median age of 73.8 years without a clear sex difference [19], whereas both historical cohorts and national database data demonstrate mild male predominance (approximately 55-57%) [6,17]. The increasing age at presentation appears to parallel the aging population of patients with PDB overall [19].

Alkaline phosphatase as a biomarker

Serum total alkaline phosphatase (ALP) reflects osteoblastic activity and overall disease burden in PDB, but its role in predicting malignant transformation remains uncertain. Case report data suggest that rapid increases in ALP may be clinically significant. A recent longitudinal report documented malignant transformation associated with ALP rising from >800 U/L to 3100 U/L in a patient harboring dual SQSTM1 and ZNF687 mutations, suggesting that abrupt biochemical escalation may signal oncogenic progression in genetically predisposed individuals [24]. However, larger series indicate that serum ALP is not invariably markedly elevated in pagetic sarcoma and correlates more strongly with the number of skeletal sites involved than with sarcoma histologic subtype [19]. Importantly, sarcoma is not necessarily associated with very high ALP levels [19].

For baseline PDB diagnosis, total ALP demonstrates limited sensitivity (57.7%) despite reasonable specificity (88.9%), and 42% of patients with radiographic PDB may have normal ALP levels [13]. No validated biochemical threshold exists for predicting malignant transformation, and current clinical guidelines state there is insufficient evidence to support bisphosphonate therapy as a preventive strategy for neoplastic degeneration [13].

Genetic susceptibility

Genetic factors represent one of the most promising areas for future risk stratification. ZNF687 mutations, particularly the P937R variant, are strongly associated with giant cell tumor complicating PDB (GCT/PDB), with all 18 reported GCT/PDB cases in one cohort harboring this identical mutation [25]. In contrast, pagetic osteosarcoma appears less tightly linked to ZNF687 alterations, with only one of 28 cases carrying a mutation (R331W) in one study [25]. This suggests tumor-specific genetic divergence within PDB-associated neoplasms.

PFN1 mutations have emerged as a particularly important marker of malignant susceptibility. Germline heterozygous deletions or mutations in PFN1 cause early-onset, polyostotic PDB with markedly increased risk of osteosarcoma and giant cell tumor development [25,56,57]. In one Italian cohort, approximately 2% of PDB patients carried germline PFN1 deletions, and these individuals demonstrated more severe disease phenotypes [25]. Notably, allelic imbalance at the PFN1 locus was detected in 57% of sporadic pagetic osteosarcomas, further implicating this pathway in tumorigenesis [25]. The identification of PFN1 mutations may therefore carry diagnostic value in identifying individuals predisposed to malignant transformation [56].

Somatic SQSTM1 mutations further support a genetic continuum between benign PDB and sarcomagenesis. Laser capture microdissection studies have identified somatic SQSTM1 mutations in affected bone from sporadic PDB patients as well as in pagetic osteosarcoma tissue, suggesting that localized genomic events may drive progression even in the absence of germline mutations [23].

Clinical features suggesting malignant transformation

Clinically, malignant transformation should be suspected in patients with sudden escalation of bone pain that is poorly responsive to medical therapy, new focal swelling, or pathological fracture [15,16]. Radiographically, cortical destruction is a critical indicator of sarcomatous degeneration, with lesions typically categorized as lytic (most common), mixed, or sclerotic [58]. One series reported that pathologic fracture with focal osteolysis was present in 50% of patients at initial tumor presentation [55]. Sarcomatous transformation is strongly suggested when focal bone destruction extends through the cortex and is associated with a soft-tissue mass [18].

Anatomic distribution and spine considerations

Although vertebral involvement is common in uncomplicated PDB, pagetic osteosarcoma tends to spare the spine [14]. In one large series, only 1 of 41 pagetic sarcomas involved vertebrae, despite the spine being affected in 30-75% of PDB cases overall [58,59]. The most common sites for pagetic osteosarcoma remain the pelvis (27-34%), femur (19-27%), and humerus (22%), with a disproportionately high frequency of humeral involvement relative to uncomplicated PDB [6,17,58]. These findings underscore that while spinal PDB is common, malignant degeneration within the vertebrae remains distinctly uncommon, further complicating predictive modeling.

Bisphosphonate therapy and prevention

The declining incidence of pagetic osteosarcoma in the modern era has been temporally associated with the widespread use of potent bisphosphonates [18]. However, current clinical guidelines explicitly state that there is insufficient evidence to conclude that bisphosphonate therapy prevents neoplastic transformation, and such treatment is not recommended for this indication [13]. The observed decline in malignant cases may reflect reduced overall PDB prevalence rather than a direct chemopreventive effect [19].

Surveillance and future directions

Given the rarity of pagetic osteosarcoma and reliance on retrospective series, predictive modeling remains limited [13,19,54,55]. Genetic screening for SQSTM1, ZNF687, and particularly PFN1 mutations may help identify higher-risk individuals, although no formal surveillance protocols currently exist [24,56,60]. Clinicians should maintain a low threshold for advanced imaging and early biopsy when malignant transformation is suspected, as delayed diagnosis contributes significantly to poor outcomes [55].

Imaging

Malignant transformation in Paget's disease of bone (PDB) should be suspected when patients develop new, rapidly progressive, or treatment-refractory symptoms. The most common clinical triggers include sudden escalation of bone pain, local swelling, and pathologic fracture [6,15,16,55]. Unrelenting pain and tender swelling are reported in up to 85% of cases, while pathologic fracture occurs in 22-50% at presentation [6,55]. In vertebral disease, neurologic symptoms-including radiculopathy, myelopathy, or bowel/bladder dysfunction-warrant urgent evaluation; neurologic deficits are reported in approximately 18% of cases [48].

Radiographically, the most critical finding is focal cortical destruction [58]. Sarcomatous transformation is characterized by bone destruction extending through the cortex with an associated soft-tissue mass [59]. Lesions are most commonly lytic (approximately two-thirds of cases), followed by mixed and sclerotic patterns [6,48,58]. Interestingly, periosteal reaction may be absent in pagetic sarcoma, which may help distinguish it from other aggressive bone tumors [48]. Most tumors arise in sites of osteoblastic or mixed pagetic bone rather than purely lytic disease [48].

Cross-sectional imaging plays a central role. MRI is superior for evaluating marrow infiltration, epidural extension, and neural compression [13,61]. Importantly, preservation of normal fatty marrow signal within pagetic bone carries a 100% negative predictive value for excluding neoplasm [61]. CT is useful for assessing cortical destruction and mineralized tumor matrix. Current guidelines do not recommend routine CT or MRI for uncomplicated PDB but endorse their use when malignancy is suspected [13,15,18].

Bone scintigraphy is highly sensitive for determining disease extent but lacks specificity for malignancy [13,18,59]. FDG-PET demonstrates potential discriminatory value. In one series, only 33% of PDB patients demonstrated FDG uptake, suggesting possible differentiation from sarcoma [62]. However, false positives may occur in active PDB [62]. For osteosarcoma staging, FDG-PET/CT demonstrates sensitivity of 87.2% and specificity of 71.4% for characterizing malignant bone tumors and is superior to Tc-99m bone scan for detecting osseous metastases [63,64].

Definitive diagnosis requires image-guided core needle biopsy performed at the treating institution to avoid contamination of surgical planes [65]. Pathologic evaluation should include tumor size, grade, invasion, and margin assessment, with decalcification methods that preserve nucleic acids (e.g., EDTA-based solutions) preferred [65]. Biopsy should not be delayed when aggressive imaging findings are present [55]. The differential diagnosis includes metastatic carcinoma, giant cell tumor, lymphoma, and infection [18,42,66,67].

Comprehensive staging for suspected pagetic osteosarcoma includes contrast-enhanced MRI ± CT of the primary site, chest CT, FDG-PET/CT or bone scan, laboratory studies (ALP, LDH), and consideration of genetic consultation [65].

Symptoms

Malignant transformation of PDB, most commonly osteosarcoma, tends to be symptomatic, especially when there is spinal involvement [6,17]. The most prevalent symptom in these cases is back pain, which is often described as a deep ache that peaks at night [68-71]. Pathologic fractures may also occur, although those can also be seen in spinal PDB without neoplasms [10,44,72]. Neurologic issues are also common and can range from radiculopathy to myelopathy with autonomic dysfunction [7,68-70]. Neurologic symptoms become most prominent after invasion of the sarcoma into the vertebral body or extension to the epidural space, resulting in compression of the neural elements [18]. Systemic manifestations, such as fever and weight loss, are uncommon with malignancies secondary to PDB but may occur in advanced disease states [7]. As with many spinal malignancies, the timing of onset is variable, which reflects the inherently aggressive nature of osteosarcoma and related neoplasms [7,25] (Table 1).

**Table 1:** Clinical and imaging features suggestive of malignant transformation in Paget's disease of bone

Category	Key findings	Supporting evidence
Clinical triggers	Sudden increase in pain, tender swelling, pathologic fracture	[6,15,16,55]
Neurologic symptoms (spine)	Radiculopathy, myelopathy, bowel/bladder dysfunction	[24,48]
Radiographic red flags	Focal cortical destruction, soft tissue mass, osteolysis	[58,59]
Lesion pattern	Lytic (most common), mixed, sclerotic	[6,48,58]
Periosteal reaction	Often absent	[48]
MRI	Marrow infiltration, epidural extension; preserved fat signal excludes malignancy	[13,61]
Bone scan	High sensitivity, low specificity	[13,18,59]
FDG-PET	Useful for staging; may differentiate benign PDB from sarcoma	[62-64]
Biopsy	Image-guided core biopsy at treating center	[55,65]

Diagnosis

For cases of PDB in which malignant transformation is suspected, especially within the spine, magnetic resonance imaging or computed tomography (CT) are typically used for initial diagnosis [42]. CT is preferred in cases in which there is a need to delineate cortical destruction and mineralized tumor matrix [42]. Staging is also completed using either chest CT to evaluate for pulmonary metastases or positron emission tomography CT (vs. bone scan) to evaluate the entire skeleton for other bony metastatic deposits [9,42]. Magnetic resonance imaging can be used to visualize the extent of marrow, spinal canal, and soft tissue involvement [55,73,74]. A bone biopsy and additional histopathological studies may be required based on the results of the initial work-up [18]. Histologically, osteosarcoma is characterized by malignant osteoid production by atypical osteoblastic cells [14,55]. The work-up for fibrosarcoma is similar, other than the fact that histologically, it is characterized by malignant spindle cells with a herringbone pattern [75].

Spine-specific surgical and multidisciplinary management

Achieving Enneking-appropriate (EA) resection with negative margins remains the strongest predictor of survival in primary spinal osteosarcoma. A meta-analysis demonstrated that EA procedures significantly reduce local recurrence (RR 0.33) and metastasis (RR 0.39), and improve 24- and 60-month survival compared with Enneking-inappropriate (EI) resections [76]. A multicenter study of 58 spinal osteosarcomas similarly showed superior median survival after EA resection (6.8 vs 3.7 years; p=0.048), with EI procedures associated with higher local recurrence rates (p=0.001) [77]. Centralized care improves margin achievement; the Oxford Spinal Sarcoma Service reported EA margins in 90% of attempted cases, with a 2% local recurrence rate in mobile spine tumors [78].

Axial location itself is associated with inferior outcomes compared to appendicular osteosarcoma. In a Cooperative Osteosarcoma Study Group analysis of 1702 patients, 10-year overall survival was 29.2% for axial tumors versus 61.7% for limb tumors (p<0.0001) [79]. The EURAMOS-1 cohort confirmed axial site as an adverse prognostic factor (HR 1.53) [80]. However, among radically resected tumors, axial location was not independently associated with worse outcome, suggesting that margin achievability rather than tumor biology drives survival disparities [80].

Spinal osteosarcoma outcomes remain heterogeneous. A Cooperative Osteosarcoma Study Group analysis of 20 mobile spine cases reported a five-year overall survival of 60% when complete resection and chemotherapy were achieved [81]. In contrast, SEER database analyses demonstrate median overall survival of approximately 15 months and five-year survival of 16-17%, with pagetic osteosarcoma carrying particularly poor outcomes (median 0.7 years) [82,83].

Chemotherapy response in spinal osteosarcoma appears suboptimal. A Rizzoli Institute series found poor histologic response to neoadjuvant therapy, with a median tumor necrosis of 20% and no patients achieving >90% necrosis [84]. While MAP-based regimens remain standard, treatment selection in older patients requires individualized assessment. NCCN guidelines note that high-dose methotrexate is often omitted in older adults, though recent data suggest selected patients ≥40 years may tolerate and benefit from adequate dosing when treated at experienced centers [28,65,85]. Conversely, other studies in patients over 50 have shown no clear survival advantage of chemotherapy over surgery alone [86], underscoring the need for individualized decision-making.

Reconstruction following spinal tumor resection is technically demanding. Consensus recommendations favor titanium cage-based reconstruction for single-level defects and structural graft augmentation for multilevel resections [87]. Long-term follow-up after total en bloc spondylectomy reveals instrumentation failure rates exceeding 50% at over 10 years, highlighting the durability challenges of axial reconstruction [88]. Alternative strategies, including cement-based reconstruction and vascularized fibular grafting, may offer acceptable medium-term outcomes in selected patients [89,90].

Given the technical complexity and prognostic implications of margin status, management of spinal pagetic osteosarcoma should occur at specialized, multidisciplinary centers capable of coordinated surgical, oncologic, and reconstructive care [65,78].

Management

Osteosarcoma secondary to PDB is typically managed with a multidisciplinary approach, much like its non-PDB-related counterpart [91]. Management is multimodal and individualized, with systemic therapy and surgery considered according to disease extent, surgical feasibility, and patient factors [92]. Unlike with osteosarcoma of the extremities, in which patients often recover well enough from surgical resection to resume chemotherapy within two to three weeks after surgery, spinal osteosarcoma likely requires three to six weeks of surgical recovery before chemotherapy can be resumed. Given the critical role of chemotherapy in patient survival, multidisciplinary treatment teams may consider "front loading" additional (if not all) cycles of chemotherapy before surgery to avoid delays in resuming chemotherapy after complex surgery. This decision is complicated and should be revisited after each chemotherapy cycle to ensure there is no significant tumor progression compromising the local control plan.

There are limited data regarding the use of bisphosphonates in patients who develop osteosarcoma from PDB. Typically, bisphosphonates are the first-line therapy for Paget's disease of the spine [93-96]. However, there is conflicting information regarding their interaction with chemotherapy. For instance, Endo-Munoz et al. reported that zoledronic acid-induced loss of osteoclasts and reduced osteolysis in primary tumors can promote pulmonary metastasis [97]. Similarly, in a clinical trial, Piperno-Neumann et al. found that the combination of chemotherapy and zoledronic acid therapy resulted in a slightly higher incidence of osteosarcoma recurrence and metastases [98]. On the other hand, studies by Yin et al. and Yuan et al. found improved chemotherapy uptake with concurrent bisphosphonate use [99,100]. However, none of these studies specifically reviewed Paget's disease of the spine, and they therefore have limited application to the present study.

En bloc surgical resection may offer the greatest chance of survival, but this is not always a technically achievable goal given the anatomic constraints of the spinal cord/dural tube, vertebral arteries, spinal nerve roots, and surrounding viscera [100-102]. When surgery is possible, preoperative planning should include consideration of the hypervascularity of bone affected by Paget's disease, which increases the risk of intraoperative bleeding [18,103]. In these cases, pretreatment with bisphosphonates (such as intravenous zoledronic acid) may reduce vascularity and bleeding risk [18,103]. Surgical treatment of spinal osteosarcoma to achieve an appropriate resection may also require instrumented fusion to stabilize the spine [104]. This can be even more challenging than it is in the treatment of metastatic spine tumors because the resections are often more extensive in osteosarcoma, and treatment is undertaken with curative intent; therefore, the reconstruction needs to be durable. In addition, PDB inherently weakens bone integrity and may result in faulty fusion, which is another reason pretreatment with bisphosphonate may improve outcomes.

Although radiotherapy is not traditionally used in the treatment of extremity osteosarcoma, it can be used in combination with surgery for the treatment of spinal osteosarcomas in which negative margins cannot be achieved because of anatomic constraints [40,92,105]. Palliative care, physical medicine, and rehabilitation also play critical roles in helping patients through complex surgical spine treatment. Early involvement of the palliative care team is recommended, given the high symptom burden and poor prognosis. Rehabilitation needs must be tailored to the degree of neurologic impairment [7,103,106]. Patients with spinal osteosarcoma are known to have worse outcomes than those with extremity osteosarcoma; in fact, in the 8th edition of their staging manual, the American Joint Committee on Cancer provided a distinct classification system for spinal osteosarcomas to highlight their unique anatomic challenges and outcomes [106]. Given the rarity of spinal osteosarcomas even in high-volume sarcoma centers, collaboration between multiple experienced centers to prospectively collect and analyze data will be critical to improving the understanding of and best practices for management of this rare cancer, including in cases associated with PDB.

Similarly, the treatment of secondary fibrosarcoma in PDB follows a multidisciplinary approach and is associated with a poor prognosis. Notably, radiotherapy is frequently used as the primary modality, unlike with osteosarcoma, but its efficacy is limited and does not substantially improve survival [7,53].

## Conclusions

Across the included studies, malignant transformation in Paget's disease of bone was consistently reported as a rare event, occurring in less than 1% of affected individuals, with spinal involvement representing only a small fraction of these cases. When present, spinal sarcomas most frequently involved the lumbar region and predominantly affected older patients, typically in the seventh to eighth decades of life, with a slight male predominance. Osteosarcoma was the most commonly reported histologic subtype, while fibrosarcoma and other sarcomas were less frequent. Clinically, back pain was the most common presenting symptom, often accompanied by neurologic deficits in cases with epidural or vertebral body involvement. Reported outcomes were consistently poor, with historical series demonstrating five-year survival rates as low as 10-15% and mean survival ranging from several months to approximately two years. Collectively, these findings suggest that although malignant transformation in PDB is rare, spinal involvement represents a distinct and highly morbid subset associated with delayed diagnosis and unfavorable prognosis.
